# Lineage-Specific Chimerism Analysis After Allogeneic Hematopoietic Cell Transplantation in Patients with Myeloid Neoplasms: Current Evidence and Considerations in the Post-Transplant Cyclophosphamide Setting

**DOI:** 10.3390/biomedicines14050952

**Published:** 2026-04-22

**Authors:** Jan Mateusz Zaucha, Jan Maciej Zaucha, Agnieszka Piekarska

**Affiliations:** 1 Department of Hematology, Transplantology and Cell Therapy, Medical University of Gdansk, 80-214 Gdansk, Poland; jazaucha@gumed.edu.pl (J.M.Z.);; 2 Department of Hematology, Transplantology and Cell Therapy, University Clinical Center, 80-214 Gdansk, Poland

**Keywords:** allogeneic hematopoietic cell transplantation, post-transplant cyclophosphamide, PTCy, chimerism, lineage-specific chimerism

## Abstract

**Background:** Chimerism analysis is a key tool for monitoring donor-cell engraftment and the risk of relapse and graft-versus-host disease (GVHD) following allogeneic hematopoietic cell transplantation (allo-HCT). The advantage of lineage-specific chimerism assessment, and its dynamics in patients receiving post-transplant cyclophosphamide (PTCy)-based GVHD prophylaxis, remains unclear. **Objective:** This review summarizes the current state of the art on chimerism analysis in patients with myeloid neoplasms undergoing allo-HCT with PTCy, with emphasis on lineage-specific testing and modern methodologies. **Methods:** A structured literature review was conducted to assess chimerism dynamics in whole blood (WB), bone marrow, and peripheral blood (PB) subpopulations, including T-cells, CD34^+^, myeloid, B, and NK (natural killer) cells, and their association with clinical outcomes following PTCy. **Results:** Lineage-specific PB chimerism, particularly in T-cells, myeloid lineage and CD34^+^ cells, is more sensitive than WB chimerism for predicting relapse. Declining donor myeloid chimerism or persistent myeloid mixed donor chimerism (MDC) may precede hematologic relapse and provide an early signal of graft instability or ineffective graft-versus-leukemia activity. T-cell MDC has been associated with an increased risk of relapse and a lower risk of GVHD, although persistent T-cell MDC in some patients may instead indicate immune tolerance. Declining CD34^+^ donor chimerism correlates with a higher risk of relapse and inferior survival outcomes and may therefore complement measurable residual disease testing. Data regarding B-cell and NK-cell chimerism remain inconsistent, likely influenced by delayed immune reconstitution. Compared to anti-thymocyte globulin, PTCy may promote higher donor T-cell chimerism, though findings across studies are variable. Next-generation sequencing (NGS) enables more sensitive detection of microchimerism and relapse prediction. **Conclusions:** Chimerism analysis, particularly when lineage-specific and NGS-based, offers valuable prognostic insight in allo-HCT with PTCy. Further prospective studies are needed to standardize testing and guide personalized post-HCT strategies.

## 1. Introduction

Chimerism analysis, the assessment of donor and recipient genetic profiles within various cell populations, is a critical tool for monitoring the outcomes of allogeneic hematopoietic cell transplantation (allo-HCT). This technique provides valuable insights into the dynamics of engraftment and the potential risk of complications such as graft-versus-host disease (GVHD) or disease relapse [1,2,3]. Among patients with myeloid neoplasms, the utility of chimerism analysis is well established and particularly pronounced in acute myeloid leukemias (AML) due to the aggressive nature of these malignancies and their frequent reliance on allo-HCT as a potentially curative therapy.

Post-transplant cyclophosphamide (PTCy) is a prophylactic strategy against GVHD with already established efficacy in overcoming mismatches in human leukocyte antigens (HLA) which, in turn, has increased donor availability and improved clinical outcomes [4]. PTCy-based protocols leverage cyclophosphamide’s immunosuppressive properties to selectively modulate alloreactive T-cells while preserving graft-versus-leukemia (GVL) effects [5,6,7,8,9,10,11]. The interaction between PTCy, chimerism patterns in peripheral blood (PB) subpopulations, and clinical outcomes remains a subject of active investigation.

This review evaluates the current literature on chimerism analysis of patients with myeloid neoplasms undergoing allo-HCT with PTCy. By focusing on the interplay between chimerism dynamics, immune reconstitution, and clinical outcomes, this review seeks to highlight key findings, identify knowledge gaps, and propose directions for future research in this evolving field.

## 2. Chimerism Overview

Chimerism analysis is routinely performed after allo-HCT to determine donor engraftment and guide potential prophylactic or salvage strategies such as immunosuppression withdrawal and donor lymphocyte infusion (DLI) [12,13]. In addition to monitoring engraftment dynamics, chimerism analysis plays a key role in the evaluation of graft failure, allowing discrimination between primary graft failure, defined as failure to achieve initial donor-derived hematopoiesis, and secondary graft failure, characterized by loss of previously established donor engraftment. In this context, declining or absent donor chimerism provides important supportive evidence for the diagnosis of graft failure [14,15,16]. Achievement of full donor chimerism (FDC) after allo-HCT is associated with both a lower probability of relapse and longer relapse-free survival (RFS) [17,18,19,20]. Recommendations from a workshop at the 2001 Tandem Meetings of the International Bone Marrow Transplant Registry (currently Center for International Blood and Marrow Transplant Research or CIBMTR) and the American Society of Blood and Marrow Transplantation (currently the American Society of Transplantation and Cellular Therapy or ASTCT) suggested performing chimerism testing at 1 month post-HCT and then at 2–4-week intervals until stable and sustained engraftment is achieved and/or at, 3, 6, and 12 months post-transplantation [3]. Although relapses occur most commonly in the first 2 years post-HCT, the risk of relapse persists in patients beyond 2 years [21,22,23,24]. Therefore, it is warranted to monitor chimerism more frequently in the first 2 years and periodically up to 5 years in patients with a higher risk of relapse [25,26].

In order to analyze chimerism, a genomic region with enough diversity of polymorphisms to distinguish donor versus recipient genetic material needs to be identified depending on the method used and the availability of genetic markers. Three commonly used approaches for chimerism analysis include short tandem repeat PCR (STR-PCR), real-time quantitative PCR (qPCR), and next-generation sequencing (NGS). While STR-PCR and qPCR are based on targeted amplification and quantification of genetic polymorphisms, NGS-based approaches combine amplification with high-throughput sequencing, enabling more comprehensive and sensitive detection of donor–recipient genetic differences. Both qPCR and NGS typically rely on multiplex analysis of single nucleotide polymorphisms and/or insertion-deletion polymorphisms across multiple genomic regions. Thus, rather than representing fundamentally distinct categories, these methods differ primarily in their analytical platforms, sensitivity, and quantitative performance. The vast majority of laboratories use STR-PCR rather than qPCR, and only a minority of laboratories use NGS [2].

STR-PCR tests have a low sensitivity (1–6%) based on the percentage of donor cells present [13]. The sensitivity of the qPCR method is 0.01%; however, the precision is much lower than in the STR-PCR method [27]. NGS, on the other hand, offers higher precision than qPCR and has a very high sensitivity (0.0001–0.1%). In the range of 0.1–90%, NGS methodology is more accurate and more linear than qPCR and STR-PCR analysis [28]. Thanks to this, it is possible to detect mixed microchimerism (MDC < 1%) at an early stage and thus apply medical intervention to prevent the disease relapse [1,29]. As more and more laboratories start to perform NGS for HLA testing, chimerism monitoring using NGS is being increasingly adopted [2].

Chimerism can be monitored from both bone marrow (BM) and PB. The published data are not unanimous on which source of chimerism could better predict the risk of disease relapse or the emergence of GVHD. Two decades ago, chimerism analysis was recommended to be performed on PB rather than on BM as it was easier to obtain the diagnostic material [3]. Moreover, detection of MDC in BM earlier and at a higher frequency than in PB does not necessarily impart higher diagnostic utility on BM chimerism. These early changes in solely BM chimerism may reflect dynamic changes that are potentially self-correcting and may not warrant therapeutic intervention. Two studies suggest that PB chimerism has a higher predictive value for disease relapse compared to BM chimerism [30,31]. In the study conducted by Gambacorta et al., the results were not statistically significant probably due to the cohort size [31].

Another important consideration is lineage-specific chimerism, which is more sensitive than whole-blood (WB) analysis but also more resource-intensive and technically demanding [32,33,34]. Commonly evaluated subpopulations include T-cells, myeloid cells, B cells, NK (natural killer) cells, and CD34^+^ progenitors. As these lineages engraft and reconstitute at different rates following allo-HCT, assessment at a single timepoint may not adequately reflect the overall dynamics of hematopoietic recovery. In addition, practical limitations related to feasibility, optimal timing, and interpretation must be taken into account. Importantly, lineage-specific chimerism should not be interpreted in isolation; rather, serial measurements are essential to capture chimerism kinetics, differentiate transient fluctuations from clinically meaningful trends, and support informed clinical decision-making. Because lineage-specific chimerism analysis relies on immunomagnetic or flow cytometric cell sorting, several pre-analytical and analytical procedures are critical for reliable results. Peripheral blood collected in EDTA is generally preferred, as it preserves DNA integrity and is compatible with both STR-PCR and qPCR-based assays [1]. Target cell purity has not been determined but generally purity of ≥90% is recommended for lineage-specific analyses. Lower purity reflects significant contamination with other lineages and can lead to misinterpretation of MDC [26]. Most laboratories require a minimum of approximately 1 × 10^4^–1 × 10^5^ sorted cells per lineage to ensure sufficient DNA yield and analytical sensitivity [35,36]. Early after allo-HCT, lymphocyte counts are often low, particularly in patients receiving PTCy, anti-thymocyte globulin (ATG), or umbilical cord blood grafts, making early lineage-specific testing challenging or uninterpretable; in such cases, WB chimerism or myeloid-lineage analysis may be more informative until adequate lymphocyte recovery occurs. Awareness of these technical constraints is essential for appropriate timing, interpretation, and clinical integration of lineage-specific chimerism results. These technical limitations are particularly relevant for CD34^+^ cell chimerism analysis, as circulating CD34^+^ cell counts are often low, especially in the early post-transplant period or during aplasia, which may preclude reliable cell sorting and limit the feasibility of this approach in routine clinical practice.

T-cells (CD 3^+^) are present in PB at sufficient frequency to allow for high-purity isolation, making them a practical target for lineage-specific chimerism testing [3]. T-cell MDC has been associated with an increased risk of disease relapse in several clinical settings, including both myeloid and lymphoid malignancies after myeloablative (MAC) and non-myeloablative regimens [20,34,37,38,39,40,41]. A recent study by Guidotti et al. demonstrated that early T-cell chimerism assessed at day +30 in bone marrow could also help identify patients at high risk of developing severe acute GVHD (grades III–IV) [42]. Previously Balon et al. reported that early FDC in T-cells was significantly associated with a higher risk of extensive chronic GVHD development, earlier GVHD onset, and immunosuppressive treatment intensity. In contrast, patients who maintained persistent T-cell MDC had a lower incidence and reduced severity of chronic GVHD, suggesting a potential tolerance-inducing effect of T-cell MDC. More recently, Baranwal et al. demonstrated that mixed T-cell donor chimerism at day +90 following MAC with PTCy was an independent predictor of inferior RFS, highlighting the prognostic importance of T-cell chimerism in this setting [43]. The presented findings underscore the clinical importance of lineage-specific chimerism monitoring and indicate that decision driven by T-cell MDC should be well-thought-out and may not always warrant preemptive intervention, particularly when considering the potential impact on GVHD development [44]. This is particularly relevant when interpreted together with myeloid chimerism as discordance between lymphoid and myeloid compartments may refine relapse risk assessment.

Myeloid cell chimerism, primarily defined by CD33^+^ and CD15^+^ cell populations, has been evaluated in various clinical settings. In a study of patients undergoing allo-HCT for chronic myeloid leukemia (CML), increasing mixed myeloid chimerism preceded cytogenetic relapse by 2 to 12 months, supporting its role as an early predictor of disease recurrence [32]. In patients with myelofibrosis (MF), the presence of mixed myeloid chimerism after allo-HCT strongly correlated with both morphologic and molecular relapse, supporting its utility in this setting [45,46]. Similarly, in allo-HCT from haploidentical donors and umbilical cord blood (UCB) for AML and myelodysplastic neoplasms (MDS), tapering UCB chimerism less than 20% in CD33^+^ cells at day 56 was associated with significantly higher relapse rates and inferior survival outcomes [47]. Additional evidence from a retrospective cohort of 154 AML patients showed that early FDC in CD33^+^ cells (<0.2% recipient DNA within 60 days post-HCT) was associated with a significantly lower relapse risk [33]. These findings collectively highlight the prognostic significance of lineage-specific myeloid chimerism in predicting post-transplant relapse across different hematologic malignancies and transplantation settings.

There is conflicting data regarding B-cell (CD19^+^) chimerism. In the study by Yang et al., mixed B-cell chimerism was more sensitive for both graft rejection and relapse compared to T and NK-cell chimerism which remained stable for longer or their changes were less predictive of clinical outcomes [48]. In contrast, another study reported no significant correlations between B-cell MDC and an elevated risk of relapse [34]. It is noteworthy that immune reconstitution in B cells occurs even up to 2 years after allo-HCT [49]. Therefore, the utility of B-lineage chimerism is questionable, especially in the early post-transplantation period. These discrepancies may be explained by heterogeneity across studies, including differences in patient populations, conditioning intensity, graft source, and GVHD prophylaxis strategies. In addition, B-cell reconstitution is often delayed and may be further affected by prior or post-transplant therapies such as anti-CD20 antibodies [50], which can significantly influence the interpretability of early B-cell chimerism results.

In terms of NK-cell chimerism, only a few published studies investigated the predictive utility of NK chimerism. The findings are inconsistent among different patient populations. In a study by Breuer et al., monitoring of both T and NK-lineage chimerism enabled stratifying patients at risk for graft loss and suggested an algorithm for risk-based assessment at an early timepoint for proactive therapeutic intervention to stabilize the graft. The stratification was based on the degree and persistence of MDC in T and NK-cells, and the early detection of these patterns provided a rationale for tailored, risk-adapted management post-HCT [51]. On the other hand, other studies reported no predictive value of MDC or FDC in NK cell lineages [34,52,53]. The conflicting findings may also reflect variability in study design, including differences in timing of sampling, use of whole blood versus lineage-sorted analyses, and the relatively small cohorts in most studies, as well as the dynamic and context-dependent nature of NK-cell reconstitution.

Available data regarding CD34^+^ cell chimerism from different studies suggests that decreasing PB CD34^+^ donor chimerism is strongly associated with a higher cumulative incidence of relapse and lower RFS and overall survival (OS) and is more sensitive in predicting relapse than WB chimerism [34,54,55,56,57,58].

To sum up, testing lineage-specific chimerism, particularly for T-cells, myeloid lineage and CD34^+^ is more sensitive in predicting impending relapse than testing WB. However, direct comparisons between CD34^+^ and myeloid-lineage chimerism are limited, and the relative superiority of these approaches remains uncertain. The utility of chimerism testing in other cell lineages remains an open question. Key findings regarding lineage chimerism analysis are presented in Table 1, which summarizes typical monitoring timepoints; however, these should be interpreted within a longitudinal framework, as no single measurement is sufficient for clinical decision-making.

### PTCy Clinical Data, Immune Reconstitution and Chimerism

When reviewing studies analyzing chimerism, it is important to reiterate that immunological reconstitution after allo-HCT varies substantially depending on both the graft source and the GVHD prophylaxis strategy used. Immune recovery differs among BM, PB stem cell (PBSC), and UCB grafts, each of which is characterized by distinct patterns and kinetics of immune reconstitution. In general, PBSC grafts are associated with faster neutrophil and T-cell reconstitution, whereas UCB grafts typically exhibit delayed T-cell recovery but relatively higher B-cell counts, and BM grafts show slower overall lymphocyte recovery compared with PBSC grafts [65,66,67,68].

In addition, differences in immune reconstitution have been described between the two most widely used GVHD prophylaxis strategies, PTCy and ATG. In these settings, immune reconstitution occurs at different timepoints across distinct cell subsets, reflecting divergent mechanisms of protection against GVHD. Specifically, CD8+ T-cells, NKT-cells, and γδ T-cells reconstitute more rapidly following ATG, whereas CD4+ T-cells and regulatory T-cells (Tregs) recover earlier after PTCy [69,70,71].

The PTCy-based protocols are currently the standard GVHD prophylaxis after haploidentical HCT (Haplo-HCT). In a retrospective study by Sanz J et al. from the ALWP EBMT, the authors evaluated the impact of Haplo-HCT transplantation outcomes in 2200 patients with AML who received GVHD prophylaxis with PTCy. The OS was 57% and GVHD-free, relapse-free survival (GRFS) 41% [72].

Transplantations from matched unrelated donors (MUD) and mismatched unrelated donors (mMUD) are associated with a high risk of GVHD and non-relapse mortality (NRM) [73,74]. The standard in Europe for the prevention of GVHD in allo-HCT from MUD and mMUD used to be, for many years, the addition of rabbit ATG (rATG) or anti-T lymphocyte globulin (ATLG) to calcineurin inhibitor (CNI) and antimetabolite (methotrexate, MTX or mycophenolate mofetil, MMF) [75]. Following the updated recommendations, in many European centers, post-PTCy, which is currently the standard in the USA, is becoming an alternative to rATG/ATLG in the MUD and mMUD transplantations on the basis of the recently published studies [76].

A comparison of PTCy- and ATG-based prophylaxis in MUD and mMUD HCT has been reported in three studies: two retrospective studies and one prospective study [77,78,79]. Most retrospective studies have proven the superiority of PTCy over ATG. However, the prospective study did not demonstrate it. Nonetheless, PTCy proved effective in the prevention of GVHD using both MAC and reduced intensity conditioning (RIC) in patients who received allo-HCT from mMUDs, MUDs, or matched sibling donors (MSDs). PTCy-based prophylaxis was well tolerated in terms of side effects and quality of life. The more detailed results of those studies are presented in Table 2.

Another study that compared protocols using PTCy-tacrolimus-MMF vs. tacrolimus-MTX showed that PTCy-based regimen resulted in a significantly higher GRFS at one year (52.7% vs. 34.9%, *p* < 0.001). This benefit was primarily driven by lower rates of grade III–IV acute GVHD and chronic GVHD without an increase in relapse incidence (RI) or NRM [80]. More recently, a randomized phase 3 trial by Curtis et al. compared PTCy plus cyclosporin to the standard regimen of cyclosporin and methotrexate in patients undergoing allo-HCT from MSDs. The study showed a significant improvement in GRFS with PTCy (median 26.2 vs. 6.4 months; HR 0.42; *p* < 0.001), with 3-year GRFS rates of 49% vs. 14%. Grade III–IV acute GVHD at 3 months (3% vs. 10%) and severe chronic GVHD (2% vs. 13%) were also significantly lower with PTCy. Although relapse rates and OS were similar between arms, the improved tolerability and GVHD profile support the growing role of PTCy as a preferred alternative to methotrexate- and ATG-based regimens in MSD transplantation using MAC or RIC [81].

In parallel, a large EBMT registry study analyzed outcomes of MSD allo-HCT with or without ATG. Incorporation of ATG significantly improved GRFS by reducing both acute and chronic GVHD, without increasing RI. The benefit was consistent across patient subgroups, highlighting the importance of GVHD prophylaxis selection for long-term outcomes, even in the MSD setting [82].

In the studies mentioned above, chimerism was not assessed. Generally, there is little published data regarding chimerism analysis after PTCy. Most of them come from single center studies or were enclosed as supplementary information. In a study conducted by Radford et al., early MDC in WB and CD3^+^ T-cells was identified as a strong negative prognostic indicator for both RFS and OS following allo-HCT for AML and MDS. Notably, the use of PTCy-based GVHD prophylaxis was associated with a significantly lower incidence of mixed CD3^+^ T-cell chimerism compared to other GVHD prophylaxis strategies [83]. Similar results were published by Retiere C et al. In that study, CD3^+^ donor chimerism was significantly higher in the PTCy group compared to the ATG group with lower RI and longer OS. Statistical significance was not achieved probably due to the small cohort [70]. In contrast, Hoff et al. reported a higher incidence of early MDC among patients receiving PTCy-based GVHD prophylaxis, which was accompanied by a lower incidence of chronic GVHD. Importantly, PTCy was not associated with inferior OS or increased relapse risk, suggesting that the presence of early MDC in this context may not necessarily indicate adverse clinical outcomes. Unfortunately, more details about chimerism assessment, whether it was from WB or a particular cell subset were not described [84]. Bilmon I et al. analyzed 12 patients with poor-risk hematologic malignancies. Chimerism status was assessable in nine patients (T-cells or WB), including eight patients who achieved complete donor T cell chimerism and one with persistent MDC who further relapsed. A statistical analysis was not performed as the cohort was small [85].

The studies quoted above unfortunately cannot be compared as the cohorts were too different and very often the key details on chimerism data are missing.

## 3. Discussion

### 3.1. BM vs. WB vs. Lineage-Specific PB Chimerism Analysis

Chimerism analysis remains a central tool in post-transplant monitoring, providing insight into engraftment dynamics, relapse risk, and immune reconstitution. BM chimerism offers high sensitivity for detecting early donor–recipient shifts; however, these changes are often transient and may not always translate into clinically meaningful outcomes. In contrast, WB chimerism is easier to obtain and has demonstrated more consistent associations with relapse risk and survival, making it suitable for routine monitoring.

Despite this, WB chimerism lacks sensitivity for detecting early lineage-specific changes. Lineage-specific PB chimerism allows for more precise assessment of hematopoietic and immune compartments and has been shown to improve early relapse detection [32,33,34]. Importantly, because different cell populations reconstitute at distinct rates after allo-HCT, single timepoint assessments may be misleading. Therefore, chimerism should be interpreted as a dynamic parameter, and serial monitoring is essential to distinguish transient fluctuations from clinically meaningful trends.

Among individual lineages, T-cell chimerism has been extensively studied and is consistently associated with relapse risk [61,86,87]. MDC in T-cells has been linked to increased relapse probability, although its interpretation remains complex, as it may also reflect immune tolerance and a reduced risk of GVHD [42]. Therefore, clinical decisions based on T-cell MDC should be made cautiously and in the context of other parameters.

The prognostic value of T-cell chimerism may be further enhanced when interpreted together with myeloid-lineage chimerism. Myeloid chimerism represents a sensitive marker of donor stem cell engraftment and early disease recurrence. Declining donor myeloid chimerism or persistent MDC often precedes hematologic relapse. Accordingly, serial monitoring of myeloid chimerism may enable early identification of patients at increased relapse risk and support preemptive interventions, such as immunosuppression tapering, DLI or targeted therapy [1,32,33,45,46,47].

Importantly, combined assessment of T-cell and myeloid chimerism may provide additional prognostic information beyond single-lineage analysis. Differences in the kinetics of lymphoid and myeloid engraftment may improve risk stratification, as discordant patterns can reflect early disease recurrence or altered GVL activity. For example, preserved donor T-cell chimerism in the presence of declining myeloid chimerism may indicate emerging relapse despite apparent lymphoid engraftment. Therefore, integrated interpretation of lineage-specific chimerism may support closer monitoring, including minimal residual disease (MRD) reassessment, and consideration of early intervention strategies in selected patients.

CD34^+^ chimerism has also been associated with relapse risk and was shown in a study by Das et al. to outperform T-cell MDC in predicting relapse [56]. CD34^+^ cells also serve as a useful source for MRD testing, which, when combined with chimerism analysis, may improve relapse prediction. However, further data are needed to justify the routine use of CD34^+^ cell sorting as a time-consuming procedure. In addition, practical constraints, such as low circulating CD34^+^ cell counts, particularly early after allo-HCT or during cytopenic phases, may impair reliable cell isolation and limit the applicability of this approach in routine clinical practice [26]. CD34^+^ cells represent a valuable compartment for MRD assessment, and their combined evaluation with chimerism analysis may further improve relapse prediction. However, the routine use of CD34^+^ chimerism is limited by technical complexity and feasibility constraints. Moreover, although CD34^+^ chimerism appears more informative than WB chimerism, direct comparative data demonstrating superiority over sorted myeloid chimerism remain limited. Therefore, it is currently unclear whether CD34^+^ assessment should be preferred over myeloid-lineage analysis in standard monitoring.

In contrast, the clinical utility of B-cell and NK-cell chimerism remains uncertain. Available data are inconsistent, likely reflecting substantial heterogeneity across studies, including differences in sampling timepoints, lineage-sorting strategies, graft source, conditioning regimens, and GVHD prophylaxis approaches. In addition, delayed and variable immune reconstitution—particularly in B cells and NK cells—limits the reproducibility and interpretability of these findings, especially in the early post-transplant period. These limitations may be further exacerbated by treatment-related factors, such as delayed NK-cell recovery after PTCy [88] and B-cell depletion associated with anti-CD20 therapies [89] or alemtuzumab-based GVHD prophylaxis [90].

Overall, lineage-specific PB chimerism represents a more sensitive and clinically informative approach than WB analysis alone, particularly when interpreted longitudinally and in combination with other biomarkers such as MRD. However, its routine implementation must take into account technical feasibility, cost, and the need for standardized interpretation. The data on clinical utility of lineage-specific chimerism are summarized in Table 3. However, it should be emphasized that no universally validated quantitative thresholds currently exist to guide clinical interventions, and decisions should be based on serial trends, reproducibility of changes, and integration with clinical context, including MRD findings.

### 3.2. NGS Chimerism Analysis

STR-PCR remains the most widely used method for chimerism assessment, despite its lower analytical sensitivity compared with newer technologies [27,28,98]. The NGS-based approaches offer improved sensitivity and quantitative accuracy, enabling the detection of low-level MDC, including microchimerism. However, the clinical relevance of detecting very low levels of recipient DNA remains uncertain.

Haugaard et al. reported a potential association between microchimerism dynamics and relapse risk, but found limited evidence supporting the prognostic value of early FDC after HCT and minimal added benefit of lineage-sorted samples over WB analysis [29]. Notably, inconsistent detection of microchimerism in patients who ultimately relapsed, as well as incomplete reporting in patients remaining in remission, highlights important methodological and interpretative limitations. These inconsistencies may be partly attributable to differences in analytical sensitivity across methods, with NGS-based approaches offering improved detection compared with STR-PCR and qPCR.

Beyond analytical sensitivity, an additional challenge lies in the interpretation of temporal changes in MDC. While absolute thresholds remain poorly defined, dynamic changes, particularly iMC, may carry prognostic significance. In the study by Jiménez-Velasco et al., iMC in PB was observed more frequently among patients who subsequently relapsed [99]. However, available data are limited and heterogeneous, and prospective studies evaluating MDC kinetics in a standardized, time-dependent manner after HCT are still lacking.

A currently ongoing prospective study utilizing NGS-based chimerism analysis may further clarify the clinical utility of high-sensitivity monitoring of MDC dynamics in patients with AML and MDS [100].

### 3.3. PTCy Chimerism Analysis

PTCy has become a widely used GVHD prophylaxis strategy due to its selective depletion of alloreactive T-cells while sparing regulatory and memory subsets. This distinct immunologic effect influences patterns of immune reconstitution and, consequently, chimerism dynamics. Most studies have reported association of PTCy with higher donor T-cell chimerism and a lower incidence of MDC compared to ATG-based regimens, which in turn led to improved RFS and OS.

However, some studies reported increased rates of early MDC following PTCy without an associated increase in relapse or decrease in OS, suggesting that the prognostic implications of MDC may differ depending on the context. These discrepancies are likely due to variations in study design, timing of chimerism assessment, methods used (e.g., WB vs. sorted populations), and differences in transplant-related variables such as donor type or conditioning regimen [70,83,85,101].

## 4. Conclusions

Key Points:

- Chimerism analysis remains a vital tool in allo-HCT monitoring, allowing engraftment assessment, relapse prediction, and immunosuppression management.
- BM chimerism is more sensitive than WB but may reflect transient, non-predictive changes and is more invasive so less practical for frequent monitoring.
- PB lineage-specific chimerism, particularly T-cell, myeloid-lineage and CD34^+^ chimerism, offers superior predictive value for relapse compared to WB chimerism.
- The role of microchimerism and iMC in relapse prediction needs further investigation using more sensitive and accurate methods like NGS for improved detection.
- The impact of PTCy on chimerism patterns remains poorly explored, necessitating further research.
- Personalized, chimerism-guided immunosuppression strategies could improve post-transplant outcomes and require further clinical validation.

Future directions:

- Standardization of Chimerism Testing Methods
  ○ Implementing uniform methodologies, particularly with the use of high-sensitivity technique like NGS in order to improve comparability across studies.
- Lineage-Specific Chimerism Assessment
  ○ Given the superior predictive value of T-cell, CD34^+^ and myeloid cell chimerism over WB chimerism, future studies should explore their role in relapse prediction and therapy adjustment.
  ○ Additional research is needed to clarify the clinical utility of other immune cell subsets in predicting relapse and graft stability.
- Microchimerism and iMC Analysis
  ○ Investigating iMC as a relapse predictor through time-dependent analyses implementing NGS could improve post-transplant monitoring and intervention strategies.
- Impact of PTCy on Chimerism Dynamics
  ○ More comprehensive studies are needed to assess how PTCy influences chimerism patterns across different immune cell subsets with the focus on correlating post-PTCy immune reconstitution with relapse risk and graft stability.
- Integration with MRD Testing
  ○ Combining chimerism analysis with MRD assessment may enhance relapse prediction, particularly in high-risk myeloid neoplasms.
- Personalized Chimerism-Guided Immunosuppression Strategies
  ○ Future research should evaluate whether personalized immunosuppression tapering based on early chimerism trends can optimize outcomes and reduce GVHD or relapse risk.
  ○ The feasibility and safety of preemptive interventions based on lineage-specific chimerism shifts warrant further investigation.

## Figures and Tables

**Table 1:** Chimerism analysis after allo-HCT: optimal timing starting point, predictive lineages and outcomes.

Timepoint (Post-HCT) [3,59,60]	Rationale for a Specific Timepoint	Most Informative Lineage(s)	Prediction of the Clinical Outcomes
Day +30 (≈ 1 month)	early engraftment and immune reconstitution; the first window for acute GVHD biology	T-cells (CD3^+^), whole blood	Acute GVHD risk (especially grade III–IV); early engraftment stability; early relapse signals in a high-risk disease [1,3,26,40,41,42,61]
Day +56–60	stabilization of myeloid engraftment; an early detection of graft failure or ineffective GVL	Myeloid cells (CD33^+^/CD15^+^)	Relapse risk; inferior OS when the low donor myeloid chimerism persists [19,32,47,62]
Day +90 (≈3 months)	transition to adaptive immune dominance; a key checkpoint for lineage divergence	T-cells (CD3^+^), CD34^+^ progenitors	mixed T-cell chimerism predicts inferior RFS (including PTCy cohorts); early molecular relapse [43,54,55,56,57]
6 months	a period when relapse kinetics of AML/MDS often emerge; a need for immunomodulation (IS tapering, DLI)	CD34^+^ progenitors, whole blood	Relapse, OS [1,16,26,56]
12 months	long-term graft stability checkpoint	CD34^+^ progenitors, T-cells	Long-term OS/PFS; detection of late relapse or secondary graft failure [1,20,54]
First 2 years (serial monitoring)	the highest cumulative risk of relapse and GVHD	T-cells, CD34^+^ cells, myeloid cells	Relapse, chronic GVHD, survival outcomes [1,22,54]
Selected patients (up to 5 years)	late relapses possible in high-risk AML/MDS or prior MDC	CD34^+^ cells	Late relapse, long-term graft stability [63,64]

Abbreviations: allo-HCT, allogeneic hematopoietic cell transplantation; ATG, anti-thymocyte globulin; BM, bone marrow; DLI, donor lymphocyte infusion; FDC, full donor chimerism; GVHD, graft-versus-host disease; MDC, mixed donor chimerism; MRD, measurable residual disease; NK, natural killer; OS, overall survival; PB, peripheral blood; PFS, progression free survival; PTCy, post-transplant cyclophosphamide; RFS, relapse-free survival; WB, whole blood. Note: Timepoints presented in this table represent commonly used clinical checkpoints and should not be interpreted as standalone decision points. Chimerism assessment requires serial monitoring to evaluate dynamic changes over time.

**Table 2:** Comparison of HCT-related outcomes with PTCy vs. ATG as GVHD prophylaxis.

Study	Penack O et al. 2024 [79]	Penack O et al. 2024 [78]	Brissot E et al. 2024 [77]	Sanz J et al. 2024 [72]
GVHD prophylaxis	PTCy vs. ATG	PTCy vs. ATG	PTCy vs. ATG	PTCy
Type of study	Retrospective	Retrospective	Prospective	Retrospective
Patient population	2123 (PTCy, *n* = 583 (27%); rATG, *n* = 1540, (73%))	8764 (PTCy, *n* = 1039 (12%); rATG, *n* = 7725 (88%))	81 (PTCy, *n* = 44 (54%); rATG, *n* = 37 (46%))	2200
Type of donor	mMUD (9/10)	MUD	MSD (*n* = 32) and MUD (*n* = 49)	Haploidentical
Conditioning	MAC 53.9% RIC 46.1%	MAC 53.3% RIC 46.4%	RIC (FluBu2)	MAC 43% RIC 57%
NRM	2 years: 18% vs. 24.9%; (*p* = 0.028, HR 0.74)	2 years: 12.1% vs. 16.4% (*p* = 0.016, HR 0.72)	5 years: 18.6% vs. 10.8% (*p* = 0.57)	2 years: 22% (20–24%)
OS	65.7% vs. 55.7% (*p* < 0.001, HR 0.77)	73.1% vs. 65.9% (*p* = 0.001, HR 0.82)	60.3% vs. 60.5% (*p* = 0.94)	57% (55–60%)
PFS	59.1% vs. 48.8% (*p* = 0.001, HR 0.78)	64.9% vs. 57.2% (*p* < 0.001, HR 0.83)	65.9% vs. 67.7% (*p* = 0.99)	52% (50–55%)
RI	22.9% vs. 26.2% (*p* = 0.068, HR 0.82)	22.8% vs. 26.6% (*p* = 0.046, HR 0.87)	27.3% vs. 37.6% (*p* = 0.52)	26% (24–28%)
Acute GVHD grade II-IV incidence rate	29.9% vs. 32.5% (*p* = 0.11, HR 0.83) (day +100)	26.5% vs. 24.1% (*p* = 0.11, HR 0.83) (day +100)	36.4% vs. 24.3% (*p* = 0.35) (6 months post allo-HCT)	28% (26–30%) (day +180)
Chronic GVHD grade II-IV incidence rate	31.7% vs. 30.3% (*p* = 0.67, HR 0.67) (2 years post allo-HCT)	28.4% vs.31.4% (*p* = 0.012, HR 0.77) (2 years post allo-HCT)	13.6% vs. 24.3% (*p* = 0.58) (time NA)	33% (31–35%) (2 years post allo-HCT)
GRFS	2 years: 46% vs. 35.3% (*p* = 0.006, HR 0.8)	2 years: 51% vs. 45% (*p* = 0.006, HR 0.8)	5 years: 43.2% vs. 37.8% (*p* = 0.39)	41% (39–43%)

Abbreviations: allo-HCT, allogeneic hematopoietic cell transplantation; ATG, anti-thymocyte globulin; rATG, rabbit anti-thymocyte globulin; GVHD, graft-versus-host disease; PTCy, post-transplant cyclophosphamide; mMUD, mismatched unrelated donor; MUD, matched unrelated donor; MSD, matched sibling donor; HLA, human leukocyte antigen; MAC, myeloablative conditioning; RIC, reduced-intensity conditioning; FluBu2, fludarabine and busulfan (two-dose regimen); NRM, non-relapse mortality; OS, overall survival; PFS, progression-free survival; RI, relapse incidence; GRFS, graft-versus-host disease-free, relapse-free survival; HR, hazard ratio.

**Table 3:** Clinical Utility Map for lineage-specific chimerism monitoring after allo-HC//T.

Lineage	Typical Sampling Windows Post-HCT	Assay Options (Typical Sensitivity) [2,91,92,93,94]	Signals that Should Prompt Action	Key Limitations
T-cells (CD3^+^) [8,20,42,51,61,83,86,87]	Day +30, +60, +90, 6 months, 12 months; serially during first 2 years	STR-PCR (~1–5%); qPCR (~0.1–1%); NGS (<0.1%)	Increasing MDC after day +60 → intensified MRD testing, IS taper, consider DLI (if no active GVHD) Early FDC with rapid kinetics → higher acute GVHD risk (especially non-PTCy settings)	Influenced by GVHD prophylaxis (PTCy vs. ATG) Delayed recovery in UCB and PTCy settings Requires sufficient lymphocyte counts early post-HCT
CD34^+^ cells [54,55,56,57]	Day +60, +90, 6 months, 12 months; serially in AML/MDS during first 2 years	STR-PCR (~1–5%); qPCR (~0.1–1%); NGS (<0.1%)	Declining donor CD34+ chimerism → early relapse signal even if MRD negative MRD+ plus falling CD34+ chimerism → consider early intervention (IS taper, DLI, targeted therapy)	Requires cell sorting (technical complexity) Low CD34+ counts in aplasia or early post-HCT may preclude testing
Myeloid cells (CD33^+^/CD15^+^) [32,33,45,47,95,96]	Day +30, +60, +90, 6 months, 12 months	STR-PCR (~1–5%); qPCR (~0.1–1%)	Low or declining donor myeloid chimerism → graft instability or relapse risk Useful for distinguishing graft failure vs. relapse	Less sensitive than CD34+ for early molecular relapse May lag behind CD34+ changes
B cells (CD19^+^) [34,48,96]	≥3 months, 6 months, 12 months (selected cases)	STR-PCR (~1–5%); qPCR (~0.1–1%)	Rarely used for relapse decisions May help in immune reconstitution profiling or post-transplant lymphoproliferation assessment	Delayed reconstitution (especially ATG, UCB) Limited evidence for relapse prediction Interpret with caution early post-HCT
NK cells (CD56^+^) [34,51,52,53,97]	Day +30, +60, +90 (mainly research/selected clinical settings)	STR-PCR (~1–5%); qPCR (~0.1–1%)	Exploratory marker of GVL and GVHD protection May complement immune monitoring in PTCy settings	Not standardized Limited prospective outcome data Not recommended as a standalone clinical decision tool

Abbreviations: allo-HCT, allogeneic hematopoietic cell transplantation; AML, acute myeloid leukemia; ATG, anti-thymocyte globulin; DLI, donor lymphocyte infusion; FDC, full donor chimerism; GVHD, graft-versus-host disease; GVL, graft-versus-leukemia; IS, immunosuppression; MDC, mixed donor chimerism; MDS, myelodysplastic neoplasms; MRD, measurable residual disease; NGS, next-generation sequencing; PB, peripheral blood; PTCy, post-transplant cyclophosphamide; qPCR, quantitative polymerase chain reaction; STR-PCR, short tandem repeat polymerase chain reaction; UCB, umbilical cord blood. Note: The signals prompting clinical action are qualitative and should not be interpreted as fixed thresholds. In the absence of standardized cut-offs, clinical decisions should be guided by serially confirmed trends, magnitude and consistency of change, and correlation with other clinical parameters (e.g., MRD, cytopenias, GVHD status).

## Data Availability

No new data were created or analyzed in this study.

## Abbreviations

The following abbreviations are used in this manuscript:

Allo-HCT	Allogeneic Hematopoietic Cell Transplantation
AML	Acute Myeloid Leukemia
ASTCT	American Society of Transplantation and Cellular Therapy
ATG	Anti-thymocyte Globulin
ATLG	Anti-T Lymphocyte Globulin
BM	Bone Marrow
cGVHD	Chronic Graft-versus-Host Disease
DLI	Donor Lymphocyte Infusion
FDC	Full Donor Chimerism
FluBu2	Fludarabine and Busulfan (2 doses)
GRFS	GVHD-Free, Relapse-Free Survival
GVHD	Graft-versus-Host Disease
Haplo-HCT	Haploidentical Hematopoietic Cell Transplantation
HLA	Human Leukocyte Antigen
iMC	Increasing Mixed Chimerism
MAC	Myeloablative Conditioning
MDC	Mixed Donor Chimerism
MDS	Myelodysplastic Syndromes
MMF	Mycophenolate Mofetil
MRD	Minimal Residual Disease
MSD	Matched Sibling Donor
mMUD	Mismatched Unrelated Donor
MUD	Matched Unrelated Donor
NGS	Next-Generation Sequencing
NK cells	Natural Killer Cells
NRM	Non-Relapse Mortality
OS	Overall Survival
PB	Peripheral Blood
PCR	Polymerase Chain Reaction
PFS	Progression-Free Survival
PTCy	Post-Transplant Cyclophosphamide
qPCR	Quantitative Polymerase Chain Reaction
RIC	Reduced Intensity Conditioning
RFS	Relapse-Free Survival
SNP	Single Nucleotide Polymorphism
STR-PCR	Short Tandem Repeat Polymerase Chain Reaction
UCB	Umbilical Cord Blood
WB	Whole Blood
